# The mitochondrial pyruvate carrier mediates high fat diet-induced increases in hepatic TCA cycle capacity

**DOI:** 10.1016/j.molmet.2017.09.002

**Published:** 2017-09-18

**Authors:** Adam J. Rauckhorst, Lawrence R. Gray, Ryan D. Sheldon, Xiaorong Fu, Alvin D. Pewa, Charlotte R. Feddersen, Adam J. Dupuy, Katherine N. Gibson-Corley, James E. Cox, Shawn C. Burgess, Eric B. Taylor

**Affiliations:** 1 Department of Biochemistry, University of Iowa Carver College of Medicine, Iowa City, IA 52242, USA; 2 Department of Anatomy and Cell Biology, University of Iowa Carver College of Medicine, Iowa City, IA 52242, USA; 3 Department of Pathology, University of Iowa Carver College of Medicine, Iowa City, IA 52242, USA; 4 Fraternal Order of the Eagles Diabetes Research Center, University of Iowa Carver College of Medicine, Iowa City, IA 52242, USA; 5 Abboud Cardiovascular Research Center, University of Iowa Carver College of Medicine, Iowa City, IA 52242, USA; 6 Pappajohn Biomedical Institute, University of Iowa Carver College of Medicine, Iowa City, IA 52242, USA; 7 AIRC Division of Metabolic Mechanisms of Disease, The University of Southwestern Texas Medical Center, Dallas, TX 75390, USA; 8 Department of Pharmacology, The University of Southwestern Texas Medical Center, Dallas, TX 75390, USA; 9 Department of Biochemistry, University of Utah School of Medicine, Salt Lake City, UT 84112, USA; 10 Metabolomics Core Research Facility, University of Utah School of Medicine, Salt Lake City, UT 84112, USA

**Keywords:** Mitochondrial pyruvate carrier (MPC), Liver, Diabetes, Gluconeogenesis, Fibrosis, Inflammation, HOMA-IR, homeostatic model assessment of insulin resistance, ITT, insulin tolerance test, HFD, high fat diet, MPC, mitochondrial pyruvate carrier, NAFLD, non-alcoholic fatty liver disease, NASH, non-alcoholic steatohepatitis, NCD, normal chow diet, T2D, type 2 diabetes, TCA, tricarboxylic acid cycle

## Abstract

**Objective:**

Excessive hepatic gluconeogenesis is a defining feature of type 2 diabetes (T2D). Most gluconeogenic flux is routed through mitochondria. The mitochondrial pyruvate carrier (MPC) transports pyruvate from the cytosol into the mitochondrial matrix, thereby gating pyruvate-driven gluconeogenesis. Disruption of the hepatocyte MPC attenuates hyperglycemia in mice during high fat diet (HFD)-induced obesity but exerts minimal effects on glycemia in normal chow diet (NCD)-fed conditions. The goal of this investigation was to test whether hepatocyte MPC disruption provides sustained protection from hyperglycemia during long-term HFD and the differential effects of hepatocyte MPC disruption on TCA cycle metabolism in NCD versus HFD conditions.

**Method:**

We utilized long-term high fat feeding, serial measurements of postabsorptive blood glucose and metabolomic profiling and ^13^C-lactate/^13^C-pyruvate tracing to investigate the contribution of the MPC to hyperglycemia and altered hepatic TCA cycle metabolism during HFD-induced obesity.

**Results:**

Hepatocyte MPC disruption resulted in long-term attenuation of hyperglycemia induced by HFD. HFD increased hepatic mitochondrial pyruvate utilization and TCA cycle capacity in an MPC-dependent manner. Furthermore, MPC disruption decreased progression of fibrosis and levels of transcript markers of inflammation.

**Conclusions:**

By contributing to chronic hyperglycemia, fibrosis, and TCA cycle expansion, the hepatocyte MPC is a key mediator of the pathophysiology induced in the HFD model of T2D.

## Highlights

• Hepatic MPC disruption protects from hyperglycemia during long-term HFD.
• HFD increases TCA cycle metabolite pool capacity and flux.
• Hepatic MPC disruption abrogates HFD-induced TCA cycle expansion.

## Graphical abstract

**Figure:**
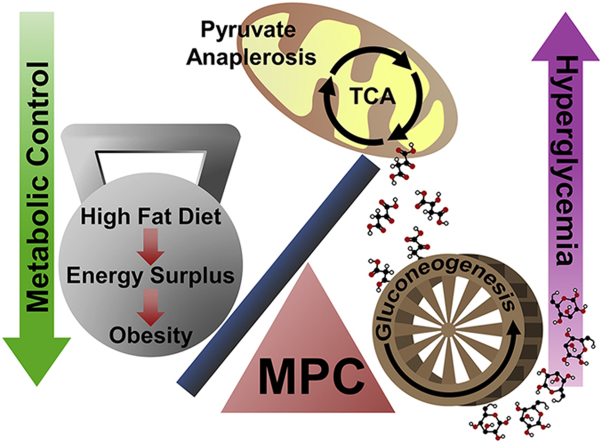

## 1 Introduction

Type 2 diabetes (T2D) is a complex disease with numerous derangements in systemic metabolism. Many result from altered function of the liver, where ectopic lipid deposition leads to insulin resistance and excessive gluconeogenesis [1], [2]. This is often accompanied by progressive liver injury, in which fatty infiltration leads to inflammation and fibrosis, that may, in-turn, contribute to systemic inflammation [3]. Although the fundamental mechanisms underlying deranged liver metabolism in T2D are not completely understood, altered mitochondrial metabolism is a prominent feature.

Hepatocyte mitochondria play a special role in systemic metabolism. During fasting, they provide energy for the whole body by supporting the synthesis of new glucose, in two key ways. They perform the initial reactions essential for channeling systemic pyruvate and amino acids into gluconeogenesis, thus gating flux. They also oxidize fatty acids to generate ATP required for gluconeogenic reactions, thereby providing essential energetic support [4], [5], [6]. However, during T2D, hepatic lipid accumulation results in loss of metabolic control, and this life-sustaining capacity becomes dysregulated and no longer suppressed by insulin. Unrestrained gluconeogenesis then drives chronic hyperglycemia, microvasculature injury, and tissue death [7]. Despite intensive investigation, the mitochondrial energetic transformations underlying excessive gluconeogenesis and how they relate to progressive liver injury are not well understood. This lack of knowledge is a fundamental barrier to designing corrective therapies.

Under most conditions, pyruvate, predominantly from systemic lactate, is the major gluconeogenic fuel [8]. The mitochondrial enzymes that shunt pyruvate towards gluconeogenesis, beginning with pyruvate carboxylase and followed by phosphoenolpyruvate carboxykinase (PEPCK), are well-studied [9]. PEPCK gates the canonical gluconeogenic pathway, but recent investigations show that factors beyond PEPCK control gluconeogenic rate [5]. Elevated flux of pyruvate through pyruvate carboxylase is consistently observed in T2D [10]. However, the mechanisms contributing to increased substrate supply to pyruvate carboxylase are unclear.

Hepatocyte mitochondrial pyruvate carrier (MPC) activity may play a fundamental role in the aberrant metabolism underlying T2D. The MPC transports pyruvate from the cytosol into the mitochondrial matrix, thereby linking glycolysis with mitochondrial metabolism [11], [12], [13]. We and others recently demonstrated that hepatocyte-specific disruption of MPC activity in vivo impairs pyruvate-driven gluconeogenesis [14], [15]. We also found that liver-specific disruption of the MPC attenuates hyperglycemia in the high fat diet (HFD) mouse model of T2D without inducing fasting hypoglycemia in normal chow diet (NCD) fed mice. However, whether MPC disruption provides sustained protection from hyperglycemia during long-term HFD, how this affects liver health, and the metabolic mechanisms underlying the differential effect on glycemia between NCD and HFD conditions were not addressed.

Here, we found that hepatocyte-specific MPC knockout (MPC LivKO) in mice results in long-term attenuation of hyperglycemia and mild protection from fibrosis. Using ^13^C-lactate/^13^C-pyruvate tracers we demonstrate that HFD increases TCA cycle capacity and that this increase is MPC-dependent. Furthermore, MPC disruption decreased levels of transcript markers of inflammation, which was recapitulated ex vivo in a primary hepatocyte model of lipotoxic inflammation. Thus, by contributing to chronic hyperglycemia, fibrosis, and TCA cycle expansion, the hepatocyte MPC is a key mediator of the pathophysiology induced in the HFD model of T2D.

## 2 Materials and methods

### 2.1 Animal use and care

All animal work was performed in accord with the University of Iowa Animal Use and Care Committee (IACUC). Two major cohorts of male mice were utilized for this study. Cohort #1 (Constitutive MPC KO cohort, n = 10) MPC LivKO mice were generated by a series of crosses between *Mpc1*^*fl/fl*^ mice and mice expressing Cre under control of the albumin promoter, as previously described [14]. At 10 weeks of age, the animals were placed into single housing with a 12-hour light: dark cycle and placed on HFD (60% kcal from fat, 20% carbohydrate, 20% protein; Research Diets Inc. #D12492i). Every 3 weeks, the animals were food restricted for 4 h (9:00AM–1:00PM) to achieve a post-absorptive state, and blood glucose and lactate levels measured. Insulin tolerance tests were performed as previously described [14]. Animals were sacrificed after a total of 44 weeks on a HFD and following 4 h of food restriction (9:00AM–1:00PM). Cohort #2 (Acute MPC KO cohort, n = 10 per final group) was placed into single housing at 7 weeks of age with ad lib water access, and mice were partitioned into normal chow (NCD; 6.5% kcal from fat, 47% from carbohydrate, 19.1% from protein; Envigo #2920i) and HFD (as above) groups. After 25 weeks of NCD and HFD treatment, MPC LivKO and control mice (WT) within each group were generated using AAV8.TBG.PI.Cre.rBG (AV-8-PV1090, AAV-Cre) and AAV6.TBG.PI.eGFP.WPRE.bGH (AV-8-PV0146, AAV-GFP), respectively, as previously described [14]. Eight weeks after AAV injection, the animals were sacrificed for the tracer metabolomics experiment detailed in Figure 3, Figure 4. Successful AAV-Cre mediated recombination of the *Mpc1*^*fl/fl*^ allele was determined after sacrifice by western blot analysis (Figure 3A).

**Figure 1:**
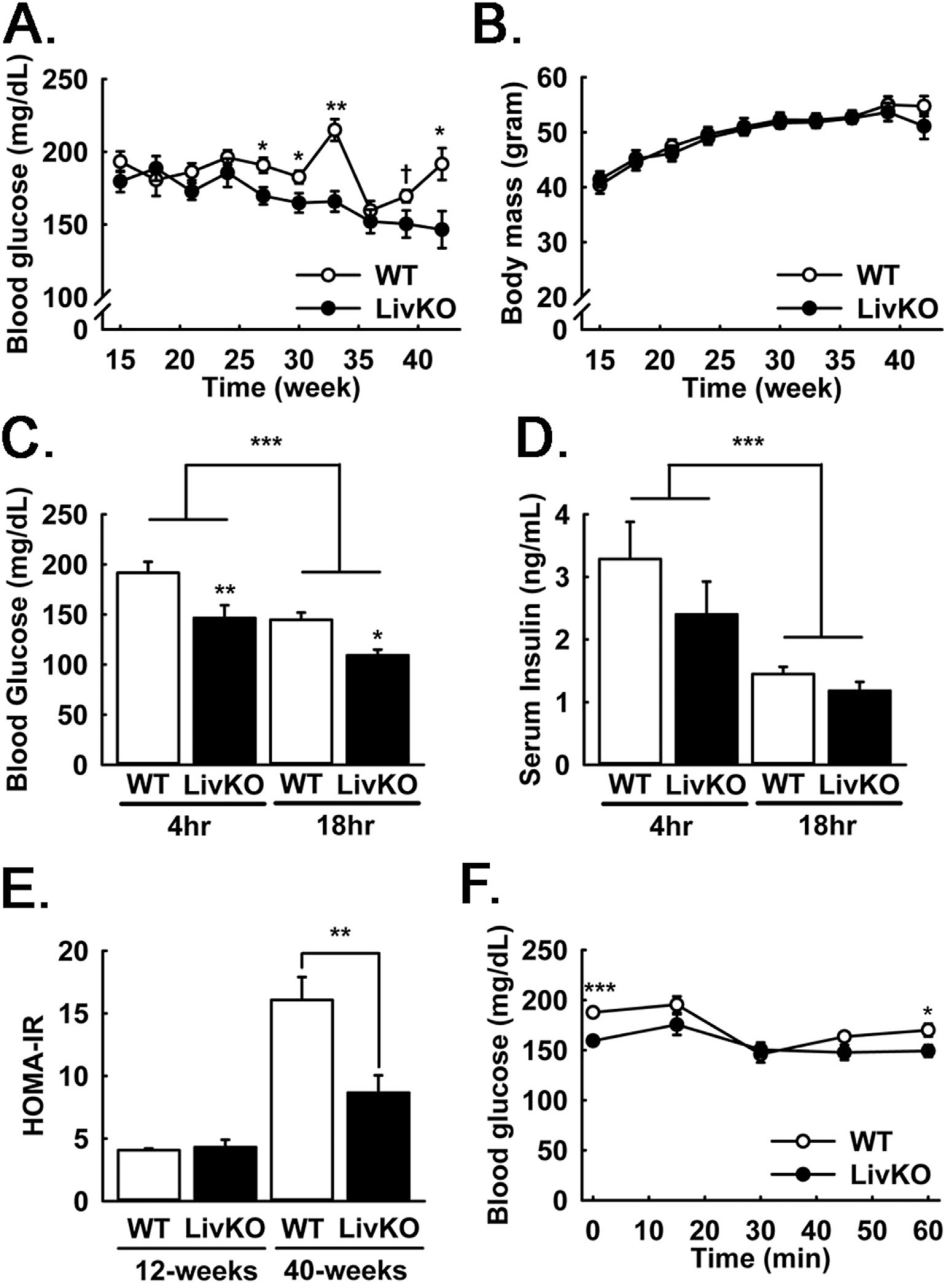
Longitudinal effects of MPC LivKO and HFD on body composition and serum parameters. (A) Serial measurements of blood glucose in WT and MPC LivKO mice. (B) Serial body mass of WT and MPC LivKO mice. (C–D) Blood glucose (C) and insulin (D) measured in WT and MPC LivKO mice following either a 4- and 18-h fast after 44 or 43 weeks of HFD, respectively. (E) HOMA-IR calculated using the 18 h fasted glucose and insulin measurements in panels C–D. (F) Insulin tolerance test performed after 32 weeks of HFD in WT and MPC LivKO mice. (Data are presented as mean ± SEM; n = 8, *p < 0.05, **p < 0.01, ***p < 0.001). Also see Figure S1.

**Figure 2:**
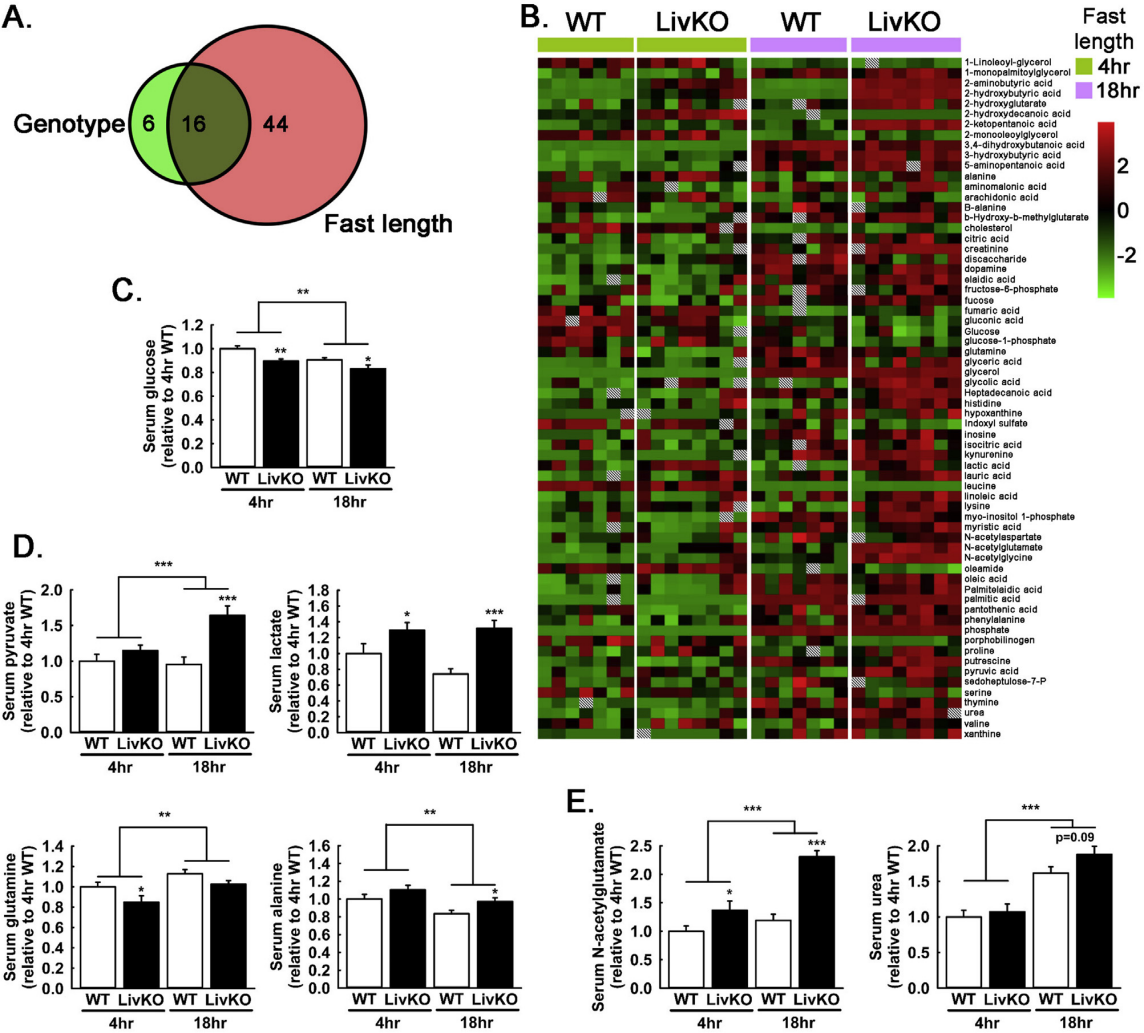
Effects of MPC LivKO and diet on the 4hr and 18hr fasted serum metabolome. (A) Venn diagram visualizing significant main effects (ANOVA) of genotype (WT and MPC LivKO, green) and fast length (4 h and 18 h, red) on the serum metabolome. A total of 66 significantly changed metabolites were identified. (B) Heat map displaying significant changes (ANOVA) in the 4 h and 18 h fasted serum metabolic profiles of WT and MPC LivKO mice. Metabolites are sorted alphabetically, with relative abundances represented on a color spectrum from red (high), to black (average), to green (low) and by hatched boxes (no value). (C) Glucose in the serum metabolic profile of WT and MPC LivKO mice. (D–E) Pyruvate, lactate, glutamine, and alanine (D) and n-acetylglutamate and urea (E) in the serum metabolic profile of WT and MPC LivKO mice. (Data are presented as mean ± SEM; n = 8, *p < 0.05, **p < 0.01, ***p < 0.001). Also see Table S1.

**Figure 3:**
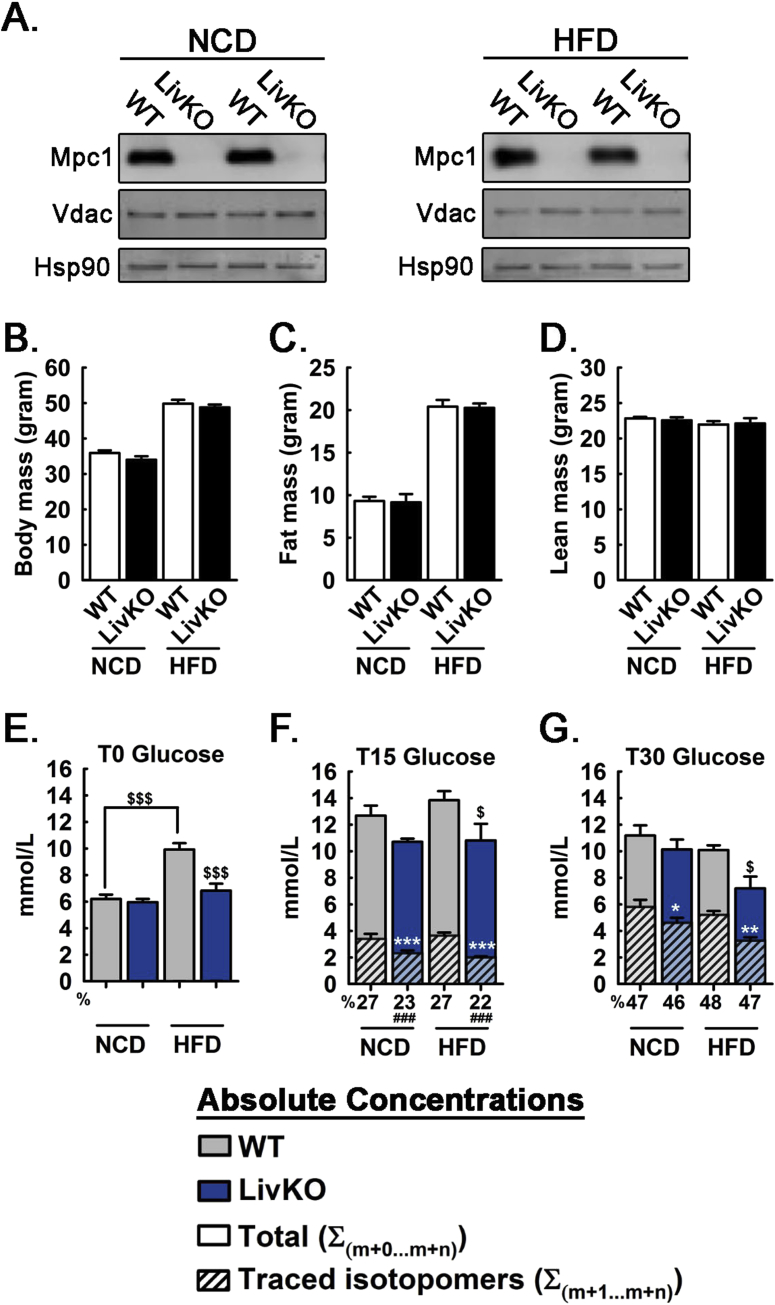
Effects of MPC LivKO and diet on serum glucose during U^13^C-lactate/U^13^C-pyruvate tolerance test. (A) Western blots of livers from AAV-GFP (WT) and AAV-Cre (LivKO) treated mice at the time of sac. Membranes were probed with antibodies detecting Mpc1, Vdac, and Hsp90. (B) Body mass of WT and MPC LivKO mice fed NCD or HFD. (C) Fat mass of WT and MPC LivKO mice fed NCD or HFD. (D) Lean mass of WT and MPC LivKO mice fed NCD or HFD. (E–G) Total traced isotopomers (hatched bars) and total pool size (solid bars) are shown for WT (gray bars) and MPC LivKO (blue bars). % ^13^C isotopomer distribution shown under the x-axis. Samples taken before injection (E), 15 min after injection (F), and 30 min after injection (G). (Data are presented as mean ± SEM; n = 5–6/genotype/diet; data analyzed by 2-way ANOVA, posthoc comparisons made using the Holm Sidak method are denoted; white significance markers compare traced isotopomers within diet *p < 0.05, **p < 0.01, ***p < 0.001; black significance markers compare total pool sizes between (as indicated) and within diets ^$^p < 0.05, ^$$$^p < 0.001; black significance markers below x-axis compare % ^13^C isotopomer distribution within diet ^###^p < 0.001). Also see Table S2.

**Figure 4:**
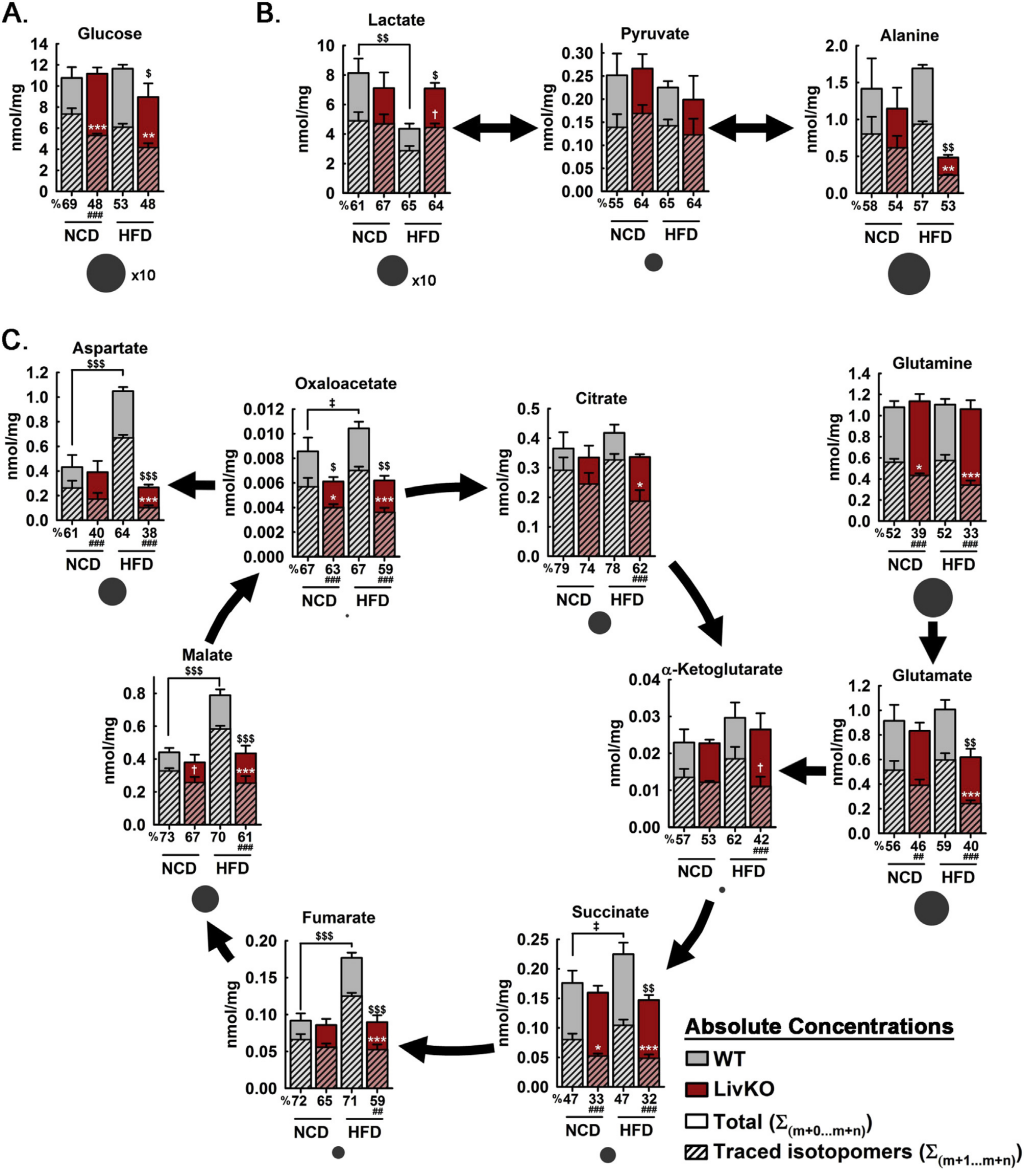
Effects of MPC LivKO and diet on hepatic TCA cycle activity during U^13^C-lactate/U^13^C-pyruvate tolerance test. (A–C) Total traced isotopomers (hatched bars) and total pool size (solid bars) are shown for WT (gray bars) and MPC LivKO (red bars). % ^13^C isotopomer distribution shown under the x-axis. Charcoal circles under bar graphs represent average relative metabolite pool size. Metabolites measured were glucose (A), Lactate, pyruvate, and alanine (B), and TCA cycle intermediates, glutamine, glutamate, and aspartate (C). (Data are presented as mean ± SEM; n = 5–6/genotype/diet; data analyzed by 2-way ANOVA, posthoc comparisons made using the Holm Sidak method are denoted; white significance markers compare traced isotopomers within diet *p < 0.05, **p < 0.01, ***p < 0.001; black significance markers compare total pool sizes between (as indicated) and within diets ^$^p < 0.05, ^$$$^p < 0.001; black significance markers below x-axis compare % ^13^C isotopomer distribution within diet ^###^p < 0.001). Also see Table S3.

### 2.2 Serum analysis

Tail vein blood was collected using capillary tubes (Sarstedt). Prior to analysis, serum was diluted to 1:3 with 0.9% saline. Aspartate aminotransferase (AST) activity assays (ThermoScientific #TR70121), Alanine aminotransferase (ALT) activity assays (ThermoScientific #TR18503), and insulin ELISA (Crystal Chem #90080) quantifications were performed using commercially available reagents according to manufacturer's directions. Calculation of HOMA-IR was performed as previously described using 18 h-fasted blood glucose and insulin levels [16], [17], [18].

### 2.3 Metabolomic analysis

Metabolomic profiling was performed as previously reported [12], [14]. Data were analyzed by two-way ANOVA (genotype and fast length). Heatmapper was used to generate a heat map of significant features [19].

### 2.4 Histology

Liver tissue was fixed in 10% neutral buffered formalin, embedded, cut at 5 μm thickness, and stained with hematoxylin and eosin (HE) or Masson's trichrome stain. All images of Masson's trichrome stain were equally color corrected in Adobe Photoshop using the “Find Dark & Light Colors” algorithm. NAFLD activity score (NAS) and fibrosis staging [20], [21] were determined from five different fields of view per mouse by a blinded observer.

### 2.5 Liver triglycerides

Liver triglycerides were extracted by the method of Folch and analyzed as previously described [14], [22].

### 2.6 Tracer metabolomics

After 33 weeks of HFD treatment, WT and MPC LivKO mice were fasted for 18 h (6:00PM–12:00PM) prior to intraperitoneal injection with a solution of 2% pyruvate and 8% lactate (10% w/v total), at a dosage of 3.0 g lactate + pyruvate/kg lean mass. 20% of both the injected pyruvate and lactate was U^13^C-labeled (Cambridge Isotopes CLM-1579 and CLM-2440). 30 min after injection, mice were anesthetized with isoflurane and a liver lobe was freeze-clamped. Tracer metabolomic analysis was conducted as previously described [14]. Traced isotopomers were calculated by multiplying the absolute concentration of isotopomers by 5, to account for the 1:4 ratio of U^13^C-labeled versus unlabeled lactate/pyruvate in the injectate.

### 2.7 Primary hepatocyte isolation and culture

Primary hepatocytes were isolated from 10-week old C57Bl/6J mice (Jackson Labs) as described previously [14]. The initial cell suspension was centrifuged at 50×*g* for 6 min, and the supernatant, enriched with Kupffer and other non-hepatocyte (non-parenchymal) cell types [23], [24], [25], was discarded. The hepatocyte-enriched pellet was then washed 2 × 3 min at 50 g to further remove debris, dead cells, and non-parenchymal cells. Hepatocytes were plated at a density of 45,000 cells/cm^2^ and allowed to attach for 4 h in Williams E media (ThermoFisher, #12551-032), supplemented with 5% FBS, 1% pen/strep, 10 nM insulin (Sigma, #I9278), and 10 nM dexamethasone (Sigma, D4902). Hepatocytes were maintained overnight in low-glucose DMEM (ThermoFisher, 11885-084) with 5% FBS and 10 nM dexamethasone. The following day, hepatocytes were treated with 300 μM palmitate conjugated to BSA, lipopolysaccharides (LPS) from Escherichia coli O111:B4 (Sigma, L2630), and 5 μM selective MPC inhibitor UK5099 (Tocris, #4186) in DMSO (<0.01% v/v final) or appropriate vehicle controls for 12 h. Following treatment, cells were washed twice with ice-cold PBS, lysed in TRIzol (ThermoFisher/Ambion), and snap frozen in liquid nitrogen for later processing.

### 2.8 qPCR

Total RNA from liver or primary hepatocyte tissue was extracted using TRIzol according to manufacturer's directions. RNA was reverse transcribed using High-Capacity cDNA Reverse Transcription Kit (Applied Biosystems) followed by qPCR reactions using SYBR Green (Life Technologies). Relative target gene mRNA abundance was normalized to 36B4 [26].

### 2.9 Data analysis

SigmaPlot or Microsoft Excel software suites were used to organize and statistically analyze data and prepare figures. Unless otherwise noted, data are represented as mean ± SEM, statistical significance was determined using a two-tailed Student's t-test or analysis of variance (ANOVA), and outliers were identified with the Grubbs test. Because the purpose of this study was to investigate the effects of MPC disruption during HFD-induced obesity, non-responders to the HFD in Cohort #1 were excluded. Non-response was defined as a percentage increase in body weight that was less than half of the group mean by 21 weeks HFD, which was the final measurement before the half-way point of the treatment. The mean weight was 55.3 ± 7.4 g and was similar for both the WT and Mpc1 LivKO mice. 2 WT and 2 Mpc1 LivKO met this criterion and were therefore excluded.

## 3 Results

### 3.1 Liver MPC disruption provides sustained protection from hyperglycemia

To investigate the role of the hepatic MPC in hyperglycemia during long-term high fat feeding, littermate *Mpc1*^*fl/fl*^ (WT) and *Mpc1*^*fl/fl*^ + Alb-Cre (MPC LivKO) mice were fed HFD for 44 weeks. Liver MPC disruption resulted in sustained attenuation of 4 h-food restricted (postabsorptive) hyperglycemia (Figure 1A). This was accompanied by slightly increased blood lactate (Figure S1A), consistent with decreased hepatic clearance and gluconeogenic utilization. Neither body mass, fat mass, nor food intake were different between WT and MPC LivKO mice throughout HFD treatment (Figures 1B, S1B, C).

To understand physiologic mechanisms for protection from hyperglycemia after 40 weeks of HFD, we measured 4 h-food restricted and 18 h-fasted blood glucose and insulin levels. Blood glucose was significantly decreased in MPC LivKO mice at both time points (Figure 1C), whereas serum insulin was decreased with fasting but not different between WT and MPC LivKO mice (Figure 1D). To evaluate the altered relationship between systemic fasting glucose and insulin levels with hepatocyte MPC disruption, we calculated HOMA-IR scores [16], [17], [18]. HOMA-IR was markedly improved in MPC LivKO mice after 40 weeks of HFD (Figure 1E). However, this effect was largely driven by the differences in 18 h-fasted glucose levels. Insulin tolerance tests (ITT) performed after 32 weeks of HFD showed minimal differences between WT and MPC LivKO mice. MPC LivKO mice showed significantly decreased blood glucose levels before insulin administration (T_0_) and at the end of the test (T_60_), but both groups responded poorly, indicative of whole-body insulin resistance (Figure 1F). Together, HOMA-IR and ITT data are consistent with decreased gluconeogenesis in MPC LivKO, versus changes in peripheral insulin sensitivity. Western blots of liver lysates confirmed loss of Mpc1 and Mpc2 proteins with MPC LivKO (Figure S1D).

### 3.2 Serum metabolomic measures of metabolic adaptation

To understand larger changes in systemic metabolism, beyond glucose and insulin levels, we examined the serum metabolomic profile of 4 h food-restricted and 18 h fasted WT and MPC LivKO mice. 106 known metabolites were detected, and statistically significant differences were observed for 66 (Figure 2A, Table S1). Significant changes in 44 metabolites were driven by fast-length alone, 6 by genotype alone, and 16 by both fast length and genotype. A depiction of overall changes by a heat-map reveals significantly changed metabolites arranged alphabetically (Figure 2B). In corroboration of measurements made with glucometers, serum glucose was decreased at both time points in MPC LivKO mice (Figure 2C). This was accompanied by increased 18 h fasted levels of pyruvate, lactate, and alanine, and decreased 4 h food-restricted glutamine (Figure 2D). Increased N-acetylglutamate was detected, and a trend towards increased 18 h fasted urea was also observed (Figure 2E). This overall pattern is consistent with decreased hepatic utilization of pyruvate, adaptively increased amino acid utilization, and consequently increased urea cycle activity.

### 3.3 Disruption of liver MPC activity mitigates HFD-induced increases in TCA cycle activity and intermediate pool sizes

We previously demonstrated that disruption of MPC activity attenuates hyperglycemia in HFD fed mice but results in minimal effects on blood glucoses levels in NCD fed mice [14]. To investigate the metabolic mechanisms leading to this differential response, we performed ^13^C-tracer experiments in NCD and HFD fed WT and MPC LivKO mice. At 7 weeks of age, littermate pairs of *Mpc1*^*fl/fl*^ mice were assigned to NCD or HFD groups, as previously described [14]. Of note, the goal of HFD treatment in this experiment was to induce obesity as a mouse model of T2D. For this purpose, we utilized a standard, non-obesogenic, phytoestrogen-free, grain-based chow diet (NCD) as a control diet. However, because a nutrient-matched, control diet was not used, this limits conclusions to effects of HFD-induced obesity, versus nutrient-specific effects, which could be important to address in future investigations.

At 32 weeks of age, mice were retro-orbitally injected with AAV-TGB-Cre to acutely disrupt *Mpc1* in hepatocytes (MPC LivKO), or AAV-TGB-GFP as a control (WT) (Figure 3A). HFD feeding continued through 40 weeks of age, and AAV-GFP and AAV-Cre groups gained weight at equal rates without differences in body composition (Figure 3B–D). At age 40 weeks, NCD and HFD fed WT and MPC LivKO mice were fasted 18 h and intraperitoneally injected with a bolus of U^13^C-lactate/U^13^C-pyruvate tracer. Dosage was normalized to lean mass, which was not different among experimental groups (Figure 3D). Delivery of a bolus models the transiently elevated macronutrient fluxes during feeding and may more strongly reveal kinetic barriers than sustained, low-level tracer infusions. Serum was sampled prior to (T_0_), 15 min after (T_15_), and 30 min (T_30_) after U^13^C-lactate/U^13^C-pyruvate injection to assess gluconeogenesis by appearance of ^13^C-glucose. Pre-injection (T_0_) blood glucose levels were significantly elevated in HFD WT mice compared to all other groups, which were not different from each other (Figure 3E, Table S2). Following tracer injection, ^13^C-enrichment into blood glucose was significantly decreased in MPC LivKO versus WT mice, in both NCD and HFD groups, and at both the T_15_ and T_30_ time points (Figure 3F,G, hatched bars).

To investigate hepatic mechanisms for decreased gluconeogenesis, liver tissue was harvested 30 min after tracer injection from live, anesthetized mice by excision of the left medial lobe and immediate freeze clamping [14], [27]. Liver tissue extracts were analyzed by mass spectrometry for glucose and TCA cycle intermediate total concentrations, ^13^C-isotopomer concentrations, and ^13^C-fractional enrichments. MPC LivKO mice in both the NCD and HFD groups displayed decreased ^13^C enrichment into hepatic glucose, indicative of decreased gluconeogenesis (Figure 4A, Table S3). Striking, reciprocal changes were observed for lactate and alanine between the HFD WT and HFD MPC LivKO groups (Figure 4B). Lactate was decreased in HFD WT mice, whereas alanine was decreased in HFD MPC LivKO mice. This result is consistent with pyruvate-alanine cycling as an MPC bypass [14], [15], [28]. It may indicate that HFD-induced increases in mitochondrial pyruvate utilization are preferentially fed from cytoplasmic lactate, with alanine serving as alternative source when MPC activity is lost.

Differences in pool sizes and ^13^C-enrichment extended to TCA cycle intermediates and other amino acids. Compared to NCD WT mice, HFD WT mice displayed increased total concentrations of aspartate, fumarate, malate, and oxaloacetate, with a trend towards increased succinate (Figure 4C, solid bars). MPC disruption in HFD WT mice decreased total TCA cycle intermediate concentrations to approximately match, or be below in the case of oxaloacetate, those in NCD WT mice. Within the HFD condition, MPC disruption significantly decreased total ^13^C-isotopomer concentrations for all measured TCA cycle intermediates, except for α-ketoglutarate, which showed decreased ^13^C-enrichment (Figure 4C, hatched bars). This combination of an unchanged total concentration with a decreased ^13^C-enrichment may indicate adaptive replenishment by amino acids. In accord, the total concentration of glutamate, which is directly deaminated to α-ketoglutarate, was decreased in HFD MPC LivKO mice. Overall, these results demonstrate HFD increases mitochondrial pyruvate utilization, and that this increase is MPC-dependent.

### 3.4 Disruption of liver MPC activity decreases HFD-induced increases in transcript markers of oxidative defense, inflammation, and fibrosis

**Figure 5:**
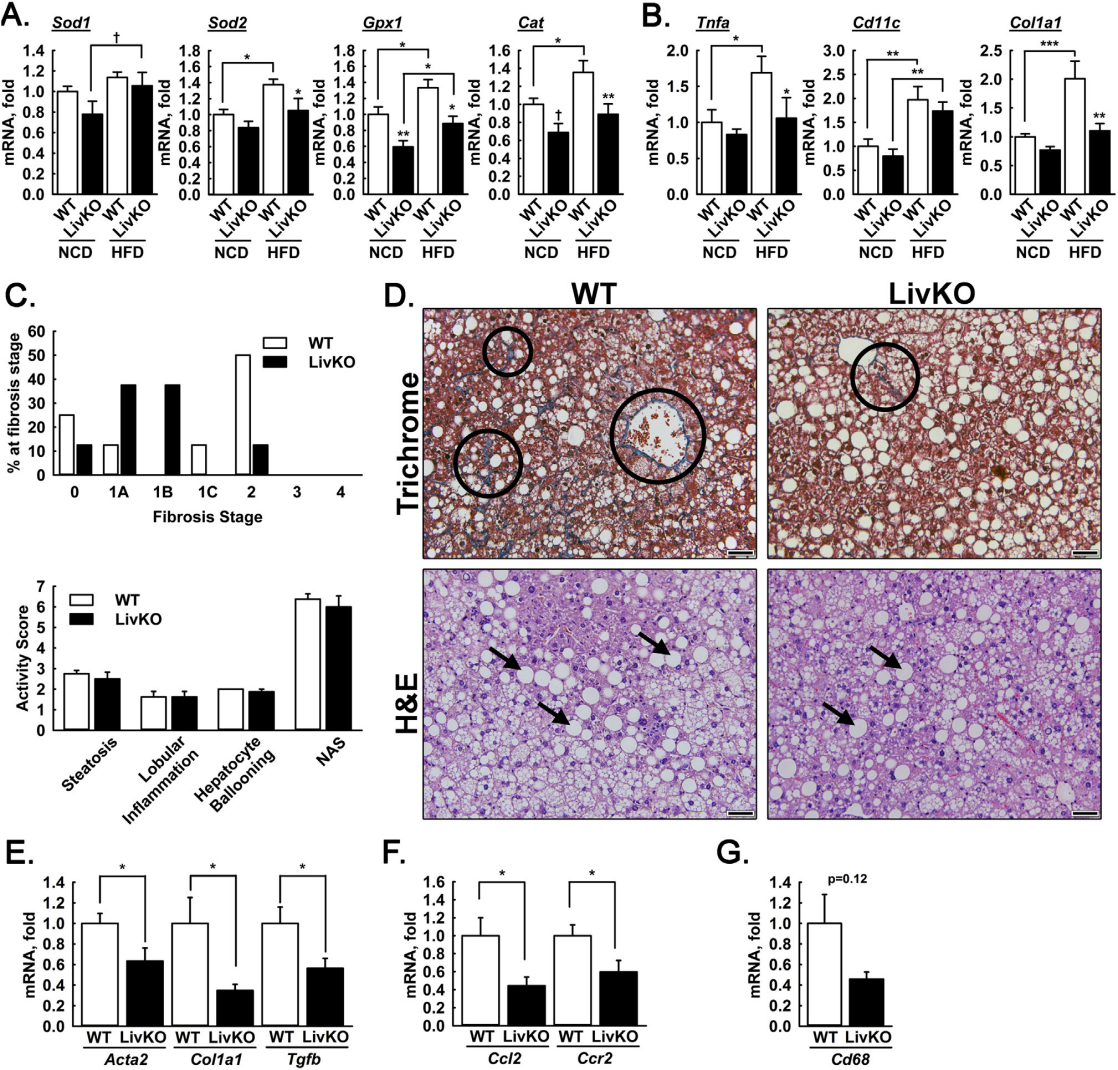
Effects of MPC LivKO and HFD on measures of liver health. (A–B) Relative liver transcript abundance of (A) oxidant defense genes (*Sod1*, *Sod2*, *Gpx1*, and *Cat*), (B) proinflammatory genes (*Tnfa*, *Cd11c*), and a fibrosis gene (*Col1a1*). (C) Fibrosis staging (upper) and NAFLD Activity Score (NAS, lower) in WT and MPC LivKO mice. Fibrosis staging is defined as: Stage 0 – no fibrosis, Stage 1A – mild perisinusoidal fibrosis, Stage 1B – moderate perisinusoidal fibrosis, Stage 1C – periportal fibrosis, Stage 2 – perisinusoidal and periportal fibrosis, Stage 3 – bridging fibrosis, and Stage 4 – cirrhosis. NAS (0–7) is the composite score of steatosis grade (0–3), lobular inflammation (0–3), and hepatocyte ballooning (0–2). A NAS greater than 5 is considered steatohepatitis. (D) Representative Masson's trichrome and H&E stained liver slices from WT and MPC LivKO mice. Arrows indicate hepatocellular vacuolation and circles indicate fibrosis (blue staining). Inset scale bar = 50 μm (E–G) Relative liver transcript abundance of (E) fibrogenic genes (*Acta2*, *Col1a1*, and *Tgfb*), (F) proinflammatory genes (*Ccl2* and *Ccr2*), (G) pan-macrophage marker (*Cd68*). (Data are presented as mean ± SEM; n = 7–8; *p < 0.05, **p < 0.01, ***p < 0.001). Also see Figure S2.

Increased TCA cycle activity may promote production of reactive oxygen species leading to oxidative damage, inflammation, and fibrosis. We examined the effects of MPC disruption on transcriptional and histological markers of liver stress, inflammation, and disease progression. In comparison to WT NCD mice, transcript levels of oxidant defense genes including mitochondrial superoxide dismutase (*Sod2*), glutathione peroxidase (*Gpx1*), and catalase (*Cat*) were increased in HFD WT but not HFD MPC LivKO mice (Figure 5A). In contrast, transcript levels of cytoplasmic superoxide dismutase (*Sod1*) were not different between HFD WT and HFD MPC LivKO mice. Decreased *Sod2* but not *Sod1* may signify a specific decrease in mitochondrial ROS. Similar to oxidant defense transcripts, the inflammatory marker *Tnfa* was increased in response to HFD feeding, which was attenuated by MPC LivKO (Figure 5B). *Cd11c*, a marker of proinflammatory macrophage polarization, was increased with HFD independent of genotype (Figure 5B). Finally, *Col1a1*, a marker of fibrosis, was increased with HFD feeding and this increase was abolished in MPC LivKO mice (Figure 5B). These results are consistent with hepatocyte MPC disruption protecting from HFD-induced increases in ROS and inflammation.

### 3.5 Disruption of liver MPC activity attenuates fibrosis during long-term high fat diet

To further examine the effects of MPC disruption on liver injury, we returned our investigation to 44 weeks HFD-fed, WT and constitutive MPC LivKO mice. We assessed histopathological severity of NAFLD stage (fibrosis) and grade (NAFLD activity score; NAS) [20], [21]. Histological fibrosis, a critical determinant in the progression of NAFLD from simple steatosis to pathogenic nonalcoholic steatohepatitis (NASH), was less severe in MPC LivKO mice (Figure 5C,D). Semi-quantitative scoring revealed that WT mice had both periportal and perisinusoidal fibrosis (Fibrosis score = 2, 4/8 mice), whereas the majority of MPC LivKO mice had either no or mild perisinusoidal fibrosis (Fibrosis score = 0 or 1, 6/8 mice). NAFLD activity score (NAS), which is a composite of steatosis, lobular inflammation, and hepatocyte ballooning quantified in H&E stained liver slices, develops readily in mice with HFD and was not different between groups (Figure 5C,D). MPC disruption did not impact liver mass to body mass ratio, liver triglycerides, or serum AST and ALT content (Figures S2A–D). Overall, these results indicate hepatic MPC disruption confers mild protection against the development of NASH during long-term HFD.

### 3.6 Disruption of liver MPC activity decreases mRNA expression signatures of inflammation

We next considered whether MPC disruption decreased hepatic inflammation as a potential mechanism for decreased fibrosis in 44 weeks HFD-fed mice. Relative mRNA abundances, measured by qPCR, of key markers of hepatic stellate cell activation (*Acta2*), fibrosis (*Col1a1*), fibrogenic stimulation (*Tgfb*), and matrix remodeling (*Mmp2*) were significantly decreased in MPC LivKO mice (Figure 5E and Figure S2E). Similarly, mRNA levels of the pro-inflammatory markers *Ccl2* and *Ccr2*, encoding the cytokine monocyte chemotactic protein-1 and its receptor, respectively, and the macrophage recruiting gene *Cyr61* were significantly decreased (Figure 5F and Figure S2F). The pan-macrophage marker, *Cd68* (p = 0.12), and the inflammasome gene *Nlrp3* (p = 0.13) also trended towards a decrease (Figure 5G and Figure S2F). Conversely, transcript levels of ATP-citrate lyase (*Acly*) and fatty acid synthetase (*Fasn*) were similar between WT and MPC LivKO (Figure S2G), indicating that changes in fibro-inflammatory state may not impinge on the lipogenic drive component of NASH progression.

### 3.7 Acute MPC inhibition in primary hepatocytes treated with palmitate and LPS

**Figure 6:**
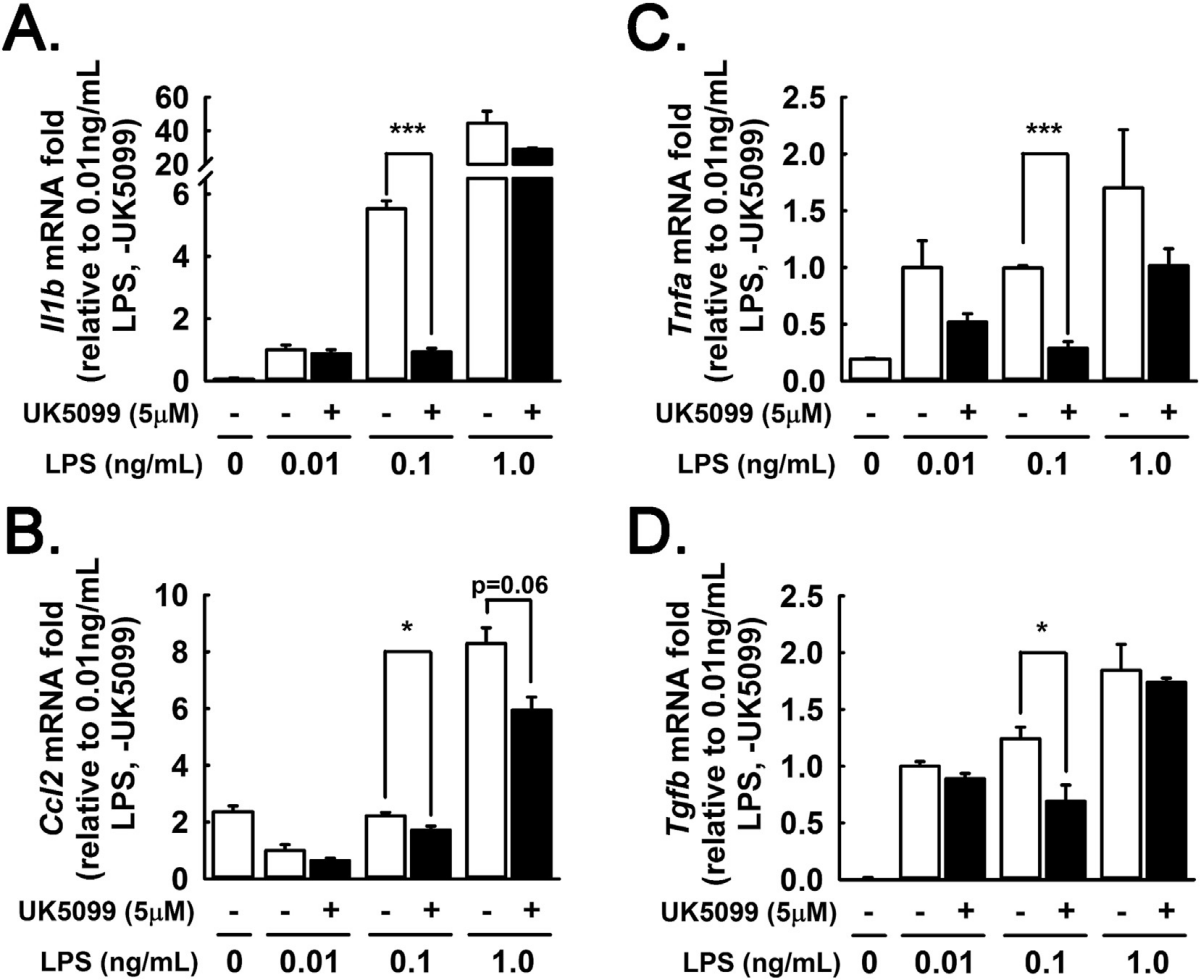
MPC inhibition attenuates inflammatory response to palmitate plus LPS. (A–E) Primary hepatocytes transcript abundance of (A) *Il1b*, (B) *Ccl2*, (C) *Tnfa*, and (D) *Tgfb* after 12hr treatment with 300 μM palmitate, LPS (0.01, 0.1, 1.0 ng/mL), and UK5099 (5 μM) or vehicle control. (Hepatocytes were pooled from three mice in technical triplicate). (Data are presented as mean ± SEM; n = 3, *p < 0.05, **p < 0.01, ***p < 0.001).

To test the acute effects of MPC inhibition on hepatocyte responsiveness to inflammatory stimuli, we implemented a primary hepatocyte model of lipotoxic inflammation. Primary hepatocytes from WT mice were pelleted with a 50×*g* centrifugation to limit co-sedimentation of Kupffer cells and other non-parenchymal cells, which pellet specifically at speeds >300×*g* [23], [24], [25]. We treated primary hepatocytes with low-dose LPS (0.01, 0.1, 1.0 ng/mL) and palmitate (300 μM conjugated to BSA), with or without the potent, selective MPC inhibitor UK5099 [29], [30]. mRNA expression of *Il1b*, *Tnfa*, *Ccl2*, and *Tgfb* exhibited a positive dose-response to increasing LPS concentration (Figure 6A–D). Chemical inhibition of MPC activity generally attenuated the induction of these genes, most significantly at the intermediate 0.1 ng/mL LPS concentration. These findings suggest that MPC inhibition increases the threshold at which hepatocytes respond to exogenous inflammatory stimuli. These data support a model where some of the anti-inflammatory effects of chronic MPC disruption in the liver in vivo persist to primary hepatocytes ex vivo and may be elicited by acute MPC inhibition. Because the primary hepatocyte isolation protocol we utilized minimizes but does not eliminate co-enrichment of non-parenchymal cells, non-hepatocyte autonomous effects cannot be ruled out.

## 4 Discussion

In prior work, we and others demonstrated that the hepatocyte MPC gates pyruvate-driven gluconeogenesis [14], [15]. We also observed that hepatocyte-specific MPC disruption exerts minimal to no effect on blood glucose in NCD fed mice but attenuates hyperglycemia in 12 weeks' HFD fed mice [14]. Here, we extend our previous work by three critical measures. First, we examine whether MPC disruption provides sustained protection from hyperglycemia during longer-term HFD treatment. Second, we evaluate how MPC disruption during long-term HFD affects liver health. Third, we investigate the metabolic mechanisms underlying the differential effects of MPC disruption on glycemia in NCD versus HFD fed mice.

Our results here show that disruption of hepatic MPC activity during 44 weeks HFD provides sustained protection from hyperglycemia. We previously found that MPC disruption evoked increased mitochondrial glutamine utilization and pyruvate-alanine cycling as adaptive mechanisms for maintenance of gluconeogenesis and euglycemia in NCD fed mice [14]. Presumably, the capacity of these mechanisms was sufficient to prevent fasting-hypoglycemia but insufficient to sustain the elevated gluconeogenesis resulting from HFD treatment. Depending on the origins of elevated gluconeogenic drive during HFD, MPC disruption during long-term HFD could lead to increased MPC-independent gluconeogenesis or compensatory gluconeogenesis from the kidneys or small intestine. If so, these adaptations could be expected to mitigate the attenuation of hyperglycemia. We did not observe this. In contrast, we observed persistent protection from hyperglycemia. This experiment was designed to investigate differences between HFD-fed WT and MPC LivKO mice and did not include an age-matched, NCD-fed reference group. As a result, the degree to which MPC disruption attenuated HFD-induced hyperglycemia cannot be fully determined. Regardless, this experiment indicates that hepatocyte MPC function is essential for some of the hyperglycemia induced by HFD.

Our results utilizing ^13^C-lactate/^13^C-pyruvate tracers demonstrate that HFD results in increased TCA cycle anaplerotic capacity. MPC disruption more potently decreased total ^13^C-isotopomer concentrations and enrichments in HFD compared to NCD mice. These findings are significant in consideration of the complex effects obesity and T2D exert on hepatic mitochondrial metabolism in humans. During insulin resistance, increased mitochondrial anaplerotic flux contributes to elevated gluconeogenesis [31]. Although impaired oxidative TCA cycle flux has been suggested as a mechanism for lipid accumulation and insulin resistance in NAFLD [32], whether or not a defect is observed probably depends on the experimental conditions and severity of the disease. Tracer approaches do not indicate impaired hepatic TCA cycle flux in lean humans with mild NAFLD (7% hepatic TAG) [33], yet demonstrate elevated TCA cycle flux in obese humans with moderate NAFLD (17% hepatic TAG) [1]. Likewise, increased β-oxidation [34], oxygen consumption [35], and elevated respiration [36] were detected in liver of obese humans. While our bolus tracer data was not intended to be fit to a steady state flux model, compared to WT NCD mice, WT HFD mice displayed greater pool sizes for several TCA cycle intermediates. MPC disruption abrogated pool size differences between NCD and HFD mice. Thus, the MPC is a key mediator of HFD-induced increases in TCA cycle anaplerotic capacity for pyruvate.

In comparison to our previous investigation [14], decreases in lactate/pyruvate flux into TCA cycle intermediates and glucose resulting from MPC disruption in NCD-fed mice were not as potent. Mice in this study were considerably older than in our previous study, which could result in differences in hepatic mitochondrial pyruvate metabolism. This is consistent with the observations that lactate-driven gluconeogenesis in rat liver slices and primary hepatocytes declines with animal age [37], [38].

Of note, administration of a lactate/pyruvate bolus results in concentrations of blood lactate [14], and presumably blood pyruvate, that are supraphysiologic except after extreme exercise. Accordingly, pool size differences observed between conditions in this experiment may not be fully representative of normal, in vivo conditions in which lactate/pyruvate delivery to the liver is markedly less and mitochondrial amino acid utilization might more proportionally compensate for MPC disruption. Nonetheless, we expect that results from this experiment are at least partially representative of normal in vivo conditions for two key reasons. First, there are numerous mechanisms by which mitochondrial metabolic control modulates TCA cycle flux independent of pyruvate supply. This point is supported by greater pyruvate utilization observed in WT HFD- versus NCD-fed mice. Second, hepatocyte MPC disruption consistently decreases HFD-induced hyperglycemia (Figure 1, Figure 2, Figure 3E) without apparent changes in peripheral insulin sensitivity (Figure 1F) [14].

The protective effects of MPC disruption from hyperglycemia and liver fibrosis raise the possibility of therapeutic targeting. Indeed, pioglitazone and other thiazolidinediones (TZD) are reported to inhibit pyruvate oxidation through an MPC-dependent mechanism [39], [40], [41]. A recent investigation demonstrated that a TZD-like molecule lacking PPAR-agonist activity attenuated weight gain and the development of NASH in mice placed on a high fat and cholesterol diet for 16 weeks. Pharmacologic protection from NASH was partially additive with liver-specific genetic disruption of the MPC by deletion of the *Mpc2* gene [42]. This may indicate extra-hepatic or MPC-independent effects of pharmacologic targeting, including the observed weight loss. Our investigation extends these findings by demonstrating that therapeutic effects of MPC disruption persist through 44 weeks of HFD. Furthermore, our findings link HFD-induced changes in TCA cycle activity with fibrosis and inflammation.

While the major purpose of this study was to examine the relationship among HFD, mitochondrial pyruvate utilization, and MPC function, our observations suggest that MPC disruption decreases oxidative stress and the downstream induction of inflammation. This is consistent with the previous observation that increases in mitochondrial metabolism contribute to the production of reactive oxygen species causing hepatic inflammation [43]. Nonetheless, we did not perform experiments here to test a causal relationship among MPC disruption, decreased ROS production, and decreased inflammation. Moreover, whether or not the anti-inflammatory effects of MPC disruption in the liver propagate to decreased systemic inflammation is currently unknown. Interactions among hepatocyte MPC function, hepatic ROS production and inflammation, and systemic inflammation will be important to mechanistically address in future studies.

In summary, hepatocyte-specific MPC disruption prevented HFD-induced TCA cycle expansion, decreased hepatic fibrosis, and attenuated hyperglycemia. Importantly, all of these occurred without weight loss, indicative of direct effects rather than secondary consequences of leanness. Thus, the hepatocyte MPC is a key mediator of T2D pathophysiology during HFD-induced obesity.

## Author contributions

AJR, LRG, RDS, SCB, and EBT designed or performed the experiments, analyzed the results, and wrote the manuscript. ADP maintained the animal colony and performed the food intake study. XF performed tracer metabolomic sample processing and data collection. JEC performed the steady state metabolomics sample processing and data collection. KNGC performed histology. CRF and AJD assisted with experiments assessing transcriptional markers of inflammation.
