# Supplementary material for: The mitochondrial pyruvate carrier mediates high fat diet-induced increases in hepatic TCA cycle capacity
Source: Mol Metab. 2017 Sep 18;6(11):1468–79. doi: 10.1016/j.molmet.2017.09.002 (PMC5681281; doi:10.1016/j.molmet.2017.09.002)
Supplement: Supplementary file 1 [file mmc1.pdf]

Table S1, related to Figure 2. Relative steady-state abundance metabolites in MPC LivKO versus WT 4hr and 18hr fasted serum. Data are presented as values ± sem, the fold change (LivKO/WT) and the associated p-value. Fold change (LivKO/WT) filled red denotes a significant increase in LivKO abundance relative to WT, whereas green fill shows a significant decrease in LivKO abundance relative to WT. p-values shown in a light red box with dark red text denote a significant difference. (Sheet1, Serum 4hr) Steady-state abundance of metabolites in 4hr fasted MPC LivKO and WT serum. (Sheet2, Serum 18hr) Steady state abundance of metabolites in 18hr fasted MPC LivKO and WT serum. (Sheet3, Serum 4hr 18hr ANOVA) ANOVA analysis of 4hr and 18hr fasted serum metabolite abundance. Significant main effects of Genotype, Diet, or the Interaction (Genotype x Diet) are shown. p-values shown in a light green box with dark green text denote $p<0.1$; and p-values shown in a light red box with dark red text denote $p<0.05$.

Table S2, related to Figure 3. $U^{13}C$ Lactate/ $U^{13}C$ pyruvate flux labeling of serum glucose. Isotopomer-distribution, -concentration, and ANOVA statistical comparison. (Sheet1, Traced Isotopomer Dist-Conc) Absolute concentration, isotopomer distribution, and traced isotopomer concentration of glucose prior to $U^{13}C$ Lactate/pyruvate injection, and after 15 or 30 minutes after $U^{13}C$ Lactate/pyruvate injection in MPC LivKO versus WT livers fed NCD or HFD. Traced isotopomer was determined by multiplying the % distribution of an isotopomer x the absolute metabolite concentration x 5 (adjustment to correct for a 20% contribution of tracer to total bolus injection). Data are presented as mean ± SEM, T-Test p-value, and the fold change (LivKO/WT) for NCD and HFD. T-Test p-values shown in a light green box with dark green text denote $p<0.1$; and p-values shown in a light red box with dark red text denote $p<0.05$. (Sheet2, ANOVA p-values) ANOVA analysis of traced isotopomers (∑m+1…m+n), total metabolite pool (∑m0…m+n), and Isotopomer distribution.

Table S3, related to Figure 4. Liver $U^{13}C$ Lactate/pyruvate flux metabolite distribution, concentration, and ANOVA statistical comparison. (Sheet1, Traced Isotopomer Dist-Conc) Absolute concentration, isotopomer distribution, and traced isotopomer concentration of metabolites 30 minutes after ^{13}C-pyruvate/^{13}C-lactate injection in MPC LivKO versus WT livers fed NCD or HFD. Traced isotopomer was determined by multiplying the % distribution of an isotopomer x the absolute metabolite concentration x 5 (adjustment to correct for a 20% contribution of tracer to total bolus injection). Data are presented as mean ± SEM, T-Test p-value, and the fold change (LivKO/WT) for NCD and HFD. T-Test p-values shown in a light green box with dark green text denote $p<0.1$; and p-values shown in a light red box with dark red text denote $p<0.05$. (Sheet2, ANOVA p-values) ANOVA analysis of traced isotopomers(∑m+1…m+n), total metabolite pool (∑m0…m+n), and Isotopomer distribution.

Figure S1, related to Figure 1

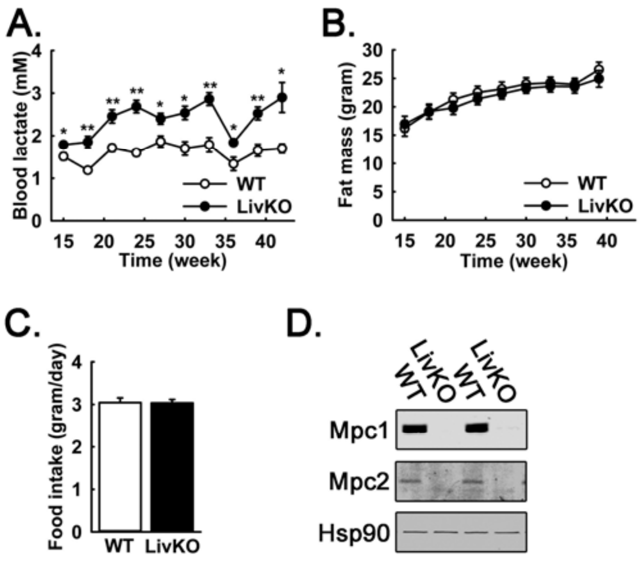

Figure S1, related to Figure 1

(A) Serial blood lactate during HFD.

(B) Serial fat mass of WT and MPC LivKO during HFD.

(C) Food intake of WT and MPC LivKO mice.

(D) Western blot analysis of Mpc1, Mpc2, and Hsp90 abundance in liver lysates. Loading was normalized to total protein, and Hsp90 is utilized as a reference protein.

(Data are presented as Mean ± SEM; n=8, $^{*}p<0.05$, $^{**}p<0.01$)

Figure S2, related to Figure 5

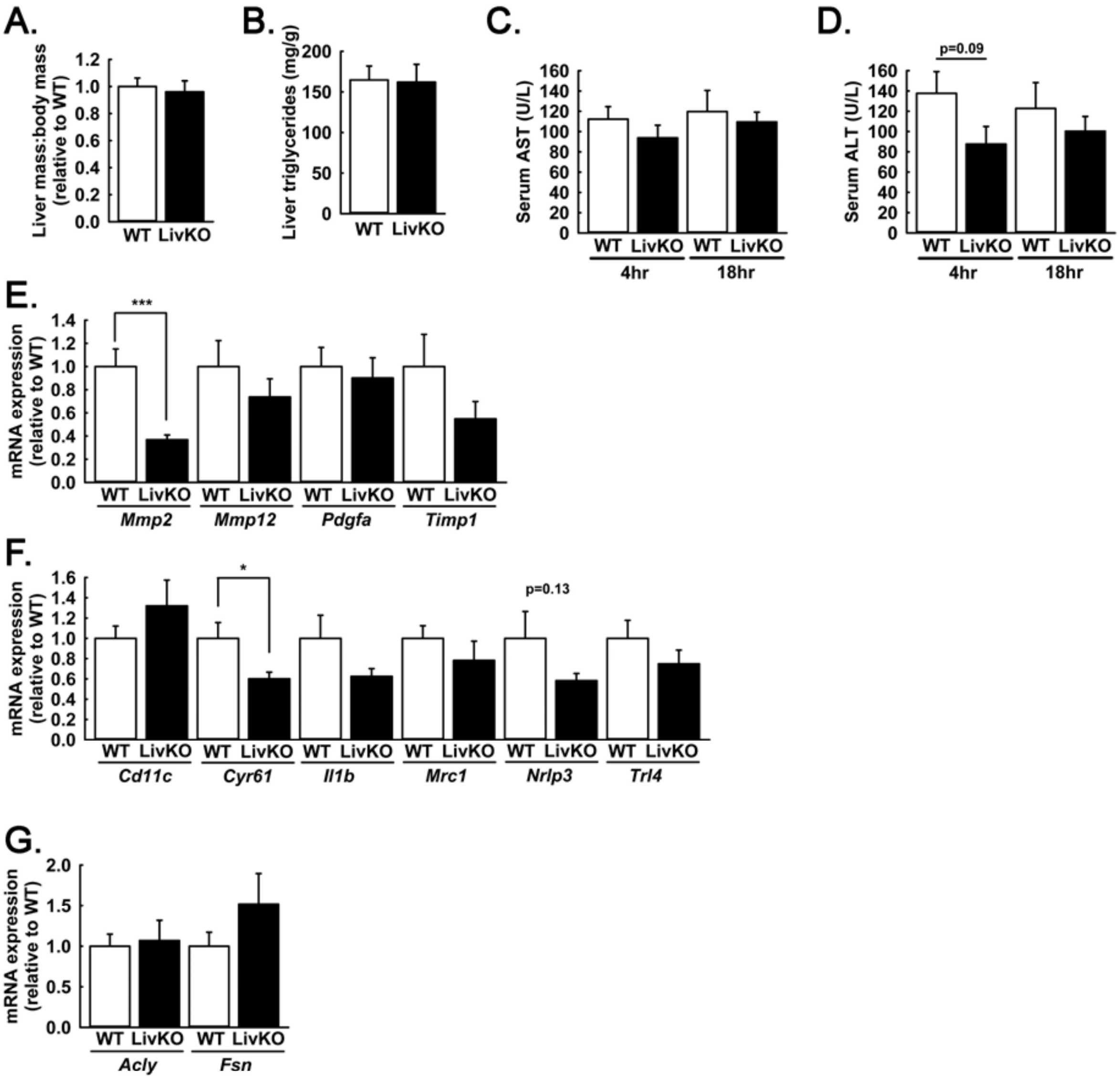

Figure S2, related to Figure 5.

(A) Liver mass:body mass was calculated for WT and MPC LivKO mice.

(B) Quantification of liver triglycerides in WT and MPC LivKO.

(C-D) Serum AST (C) and ALT (D) of 4hr and 18hr fasted WT and MPC LivKO mice.

(E-G) Relative liver transcript abundance of (E) fibrogenic genes (*Mmp2*, *Mmp12*, *Pdgfa*, and *Timp1*), (F) proinflammatory genes (*Cd11c*, *Cyr61, Il1b*, *Mrc1*, *Nrlp3*, and *Trl4*), (G) de novo lipogenic genes (*Acly* and *Fsn*), of WT and MPC LivKO mice.

(Data are presented as Mean ± SEM; n=7-8/group. *p<0.05, ***p<0.001).

EXTENDED EXPERIMENTAL PROCEDURES

Animal Use and Care

All animal work was performed in accordance with the University of Iowa Animal Use and Care Committee (IACUC). Two distinct animal cohorts of male mice were utilized for this study. Cohort #1 (Long-Term cohort, n=10) Mpc1 LivKO mice were generated by a series of crosses between *Mpc1*$^{fl/fl}$ mice and mice expressing Cre under control of the albumin promoter, as previously described (Gray et al., 2015). At 10 weeks of age the animals were placed into single housing with 12-hour light: dark cycle and placed on HFD (60% kcal from fat, 20% carbohydrate, 20% protein; Research Diets Inc.). Every three weeks the animals were food restricted for four hours to achieve a post-absorptive state and blood glucose and lactate levels measured. Insulin tolerance tests were performed as previously described (Gray et al., 2015). Animals were sacrificed after a total of 44 weeks on a HFD.

Cohort #2 (tracer cohort, n=10) was, at 7 weeks of age, placed into single housing with ad lib water access and placed on a HFD or continued on normal chow (NCD; 6.5% kcal from fat, 47% from carbohydrate, 19.1% from protein; Envigo). Mpc1 LivKO and WT mice were generated using AAV8.TBG.PI.Cre.rBG (AV-8-PV1090, AAV-Cre) and AAV6.TBG.PI.eGFP.WPRE.bGH (AV-8-PV0146, AAV-GFP) as previously described (Gray et al., 2015) after 25 weeks of HFD. 8 weeks after injection the animals were sacrificed for the Tracer Metabolomics experiment detailed in Figure 3. Successful AAV-Cre mediated recombination of the *Mpc1*$^{fl/fl}$ allele was determined after sacrifice by western blot analysis (Figure 3A).

Serum Analysis

Tail vein blood was collected using capillary tubes (Sarstedt Microvette CB300). After collection, the serum was separated via centrifugation at 3000xg for 10 minutes, flash frozen, and stored at -80°C. Prior to analysis serum was diluted to 1:3 with 0.9% saline. AST (ThermoScientific #TR70121), ALT (ThermoScientific #TR71121), and insulin (Crystal Chem #90080) quantifications were performed using commercially available reagents according to manufacturer's directions. Calculations of HOMA-IR and QUICKI were performed following an 18-hour fast as previously described (Lee et al., 2008).

Metabolomic Analysis

Serum was collected from mice after 4 and 18 hours fasting. Metabolomic profiling was performed as previously reported (Bricker et al., 2012; Gray et al., 2015).

Histology

Liver tissue was fixed in 10% neutral buffered formalin, embedded, cut at 5μm thickness, and stained with hematoxylin and eosin (HE) or Masson's trichrome stain. All images of Masson's trichrome stain were equally color corrected in Adobe Photoshop using the "Find Dark & Light Colors" algorithm.

Western Blots

Liver tissues were prepared as previously described (Gray et al., 2015). Standard SDS-PAGE and western blot protocols were followed. Proteins were size-separated by 15% SDS-PAGE gel, transferred to 0.22μm nitrocellulose membranes (GE Healthcare, #10600001), and blocked with TBST supplemented with 5% nonfat dry milk. Blots were incubated with primary antibodies at 4°C overnight and fluorescent secondary antibodies for 1 hour, and visualized using the Li-Cor Odyssey CLx system.

Antibodies

Primary Antibodies: MPC1 (1:1,000, generous gift from Dr. Brian Finck), MPC2 (1:1,000, CST #46141S), and HSP90 (1:1,000, CST #4874). Secondary antibody: Goat anti-Rabbit DyLight 800 (1:10,000, ThermoFisher #35571)

Liver Triglycerides

Liver triglycerides were extracted by the method of Folch and analyzed as previously described (Folch et al., 1957; Gray et al., 2015).

Tracer Metabolomics

After 33 weeks of HFD treatment WT and Mpc1 LivKO mice were fasted for 18 hours prior to being injected i.p. with a 10% solution of $U^{13}C$-pyruvate/$U^{13}C$-lactate (20% ^{13}C-labeled, 80% unlabeled; 20% pyruvate:80% lactate; Cambridge Isotopes CLM-1579 and CLM-2440) at 3.0mg/kg lean mass. 30 minutes after injection mice were anesthetized with isoflurane and a liver lobe was freeze-clamped. Samples were stored at -80C until they were prepared for GC-MS analysis of glucose, organic acids, and amino acids. Tracer metabolomics analysis was conducted as previously described (Gray et al., 2015). Traced isotopomers were calculated by multiplying the absolute concentration of isotopomers by 5 to correct for 1 labeled:4 unlabeled that was injected.

Primary Hepatocyte Isolation and Culture

Primary hepatocytes were isolated from 10-week old C57Bl/6J mice (Jackson Labs 000664) using the two-step collagenase perfusion method as described previously (Gray et al., 2015). Cell viability was between 80-90% as assessed by trypan blue. Cells were washed 3X (DMEM, 4.5g/L glucose, 5% FBS, 10mM HEPES pH=7.4, 1% pen/strep) and plated at a density of $3.7x10^4$ cells/cm^2 in attachment media (Williams E Media, 100nM insulin, 10nM dexamethasone, 5% FBS, 1% pen/strep) for four hours. Media was changed to experiment base media (DMEM 4.5g/L glucose, 2mM GlutaMAX, 100nM insulin, 10nM dexamethasone, 5% FBS) overnight. Cells were then treated with 300μM palmitate conjugated to BSA, 0.01, 0.1 or 1.0ng/mL LPS (Sigma), and 5μM selective MPC inhibitor UK5099 (Sigma) or appropriate vehicle controls for 12 hours. Following treatment, cells were washed twice with ice-cold PBS, lysed in TRIzol (ThermoFisher/Ambion #15596026), and snap frozen in liquid nitrogen for later processing.

qPCR

Total RNA from liver tissue was extracted using TRIzol according to manufacturer's directions. For each sample an equal amount of RNA was reverse transcribed using High-Capacity cDNA Reverse Transcription Kit (Applied Biosystems #4368814) followed by qPCR reactions using SYBR Green (Life Technologies #4309155). Relative abundance of mRNA was normalized to 36B4 (Akamine et al., 2007).

Primer sequences: 5'-3'

Acyl-Forward	AGGAAGTGCCACCTCCAACAGT
Acyl-Reverse	CGCTCATCACAGATGCTGGTCA
Acta2-Forward	GTACCACCATGTACCCAGGC
Acta2-Reverse	GCTGGAAGGTAGACAGCGAA
Cat-Forward	ACAAGATTGCCTTCTCCGGG
Cat-Reverse	AGGACATCAGGTCTCTGCGA
Cd11c-Forward	ACACTGAGTGATGCCACTGTC
Cd11c-Reverse	TTTAACTCGAGTCAGAGCCCG
Cd68-Forward	GTGTCTGATCTTGCTAGGACC
Cd68-Reverse	GTGCTTTCTGTGGCTGTAG
Ccl2-Forward	TGCCCTAAGGTCTTCAGCAC
Ccl2-Reverse	AAGGCATCACAGTCCGAGTC
Ccr2-Forward	AGTTCAGCTGCCTGCAAAGA
Ccr2-Reverse	GCCGTGGATGAACTGAGGTA
Col1a1-Forward	CAGTCGCTTCACCTACAGCA
Col1a1-Reverse	CGGGAGGTCTTGGTGGTTTT
Cyr61-Forward	GTTCCTAGTGTGGGCTGGAC

Cyr61-Reverse	AAGGCACCATTCATCCTCCG
Fasn-Forward	CGGTAGCTCTGGGTGTA
Fasn-Reverse	TGCTCCCA GCTGCAGGC
Gpx1-Forward	CGGCACAGTCCACCGTGTAT
Gpx1-Reverse	CATTCTCCTGGTGTCCGAACTG
Il1b-Forward	GCCACCTTTTGACAGTGATGAG
Il1b-Reverse	AGCTTCTCCACAGCCACAAT
Mcr1-Forward	GGAGGGTGCGGTACACTAAC
Mcr1-Reverse	CAGGCAGTTGAGGAGGTTCA
Mmp2-Forward	ACGATGATGACCGGAAGTGG
Mmp2-Reverse	GTGTAGATCGGGGCCATCAG
Mmp12- Forward	TGAATTTGCTGAATGGTACTTGTC
Mmp12-Reverse	CCCAGTTGCTTCTAGCCCAA
Nlrp3-Forward	ATGGCTGTGTGGATCTTTGC
Nlrp3-Reverse	CACGTGTCATTCCACTCTGG
Pc-Forward	CAGGAGAACATCCGCATCAAT
Pc-Reverse	CTCACCACTCCGGAAAACCT
Pck1-Forward	CGGAAGAGGACTTTGAGAAAGCATTC
Pck1-Reverse	GCGAGTCTGTCAGTTCAATACCAATC
Pdgfa-Forward	TGTAACACCAGCAGCGTCAA
Pdgfa-Reverse	TCTCACCTCACATCTGTCTCCT
Tgfb1-Forward	AAATCAACGGGATCAGCCCC
Tgfb1-Reverse	CGCACACAGCAGTTCTTCTC
Tnfa-Forward	AGAGACTTTAGGCCTCCGCA
Tnfa-Reverse	AGCCTGATTTGGTGACCAGG
Timp1-Forward	TCTCTGGCATCTGGCATCCT
Timp1-Reverse	TCTCGTTGATTTCTGGGGAACC
Tlr4-Forward	TGGCTGGTTTACACGTCCAT
Tlr4-Reverse	TGCAGAAACATTCGCCAAGC
Tnfα-Forward	GACAAGCCTGTAGCCCACG
Tnfα-Reverse	TGTCTTTGAGATCCATGCCGT
Sod-Forward	AAGAGAGGCATGTTGGAGACC
Sod-Reverse	CGGCCAATGATGGAATGCTC
Sod2-Forward	GGAGCAAGGTCGCTTACAGA
Sod2-Reverse	CAGCGGAATAAGGCCTGTTGT
36B4-Forward	CGTCCTCGTTGGAGTGACA
36B4-Reverse	CGGTGCGTCAGGGATTG

Data Analysis

SigmaPlot or Microsoft Excel software suites were used to organize and statistically analyze data and prepare figures. Unless otherwise noted, data are represented as mean ± SEM, statistical significance determined using a two-tailed Student's t-test or analysis of variance (ANOVA as appropriate), and outliers were identified with the Grubbs test. Because the purpose of this study was to investigate the effects of MPC disruption during HFD-induced obesity, non-responders to the HFD in Cohort #2 were excluded. Non-response was defined as a percentage increase in body weight that was less than half of the group mean by 21 weeks HFD, which was the final measurement before the half-way point of the treatment. The mean weight was 55.3 ± 7.4 grams and was similar for both the WT and Mpc1 LivKO mice. 2 WT and 2 Mpc1 LivKO met this criterion and were therefore excluded.
